# “Ain’t She a Bute?”: The Importance of Proper History Taking in a Case of Inappropriate Use of Horse NSAID in a Human

**DOI:** 10.3390/clinpract11030060

**Published:** 2021-07-08

**Authors:** Khalid Sawalha, Ryan James, Farah Mazahreh, Harmeen Goraya, Fuad Habash

**Affiliations:** 1 Internal Medicine Division, White River Health System, Batesville, AR 72501, USA; 2 Internal Medicine Division, University of Arkansas for Medical Sciences, Little Rock, AR 72205, USA; rjames@uams.edu (R.J.); fmazahreh@uams.edu (F.M.); 3 Pulmonary and Critical Care Division, University of Arkansas for Medical Sciences, Little Rock, AR 72205, USA; hgoraya@uams.edu; 4 Cardiology Division, University of Arkansas for Medical Sciences, Little Rock, AR 72205, USA; FHabash@uams.edu

**Keywords:** NSAID, phenylbutazone, Bute, NSAID toxicity, toxicity

## Abstract

A 41-year-old woman with no significant past medical history presented to the hospital with complaints of nausea, vomiting, and generalized weakness over two weeks. The patient did not seek medical attention as she assumed that her symptoms willwould resolve. Following her initial denial of drug abuse and her abnormal urine drug screening, we discussed the findings with the patient. She later admitted to using both amphetamines and marijuana. This led us to take a detailed social history that revealed an unexpected event.

## 1. Introduction

A patient’s history and physical examination (H&P) acts as one of the foundations of delivering effective healthcare to our patients; however, due to patient load and time constraints, it can often be tempting to take shortcuts or rely on the history obtained from others. As the pace of delivering care continues to rise and decreased patient healthcare literacy continues to be a barrier, the H&P is more important now than ever. This case report shows the importance of taking a proper history to not only expedite diagnosis and treatment but to establish a trusting relationship between patient and physician.

## 2. Case Presentation

A 41-year-old woman with no significant past medical history presented to the hospital with complaints of nausea, vomiting, and generalized weakness over two weeks. The patient was doing well prior to her presentation and she assumed that her symptoms would resolve without seeking medical attention. The patient reported subjective fevers with severe back pain during this period. The patient often had mild back pain; this is usually controlled by naproxen over the counter but provides little relief during this episode. The patient felt progressively worse and lost her appetite; thus, her husband suggested drinking large volumes of fluids. The patient reported drinking a gallon of cranberry juice, six 16oz bottles of water, and four 12oz cans of Sprite, leading to severe diarrhea and further weakness. The patient reported that she had a tick bite around the time of her initial symptoms which stayed on her skin for an hour. She avoided seeking medical attention as this was a normal occurrence given where she lives in the country. The patient denied smoking or illegal drug abuse.

On examination, the patient was tachycardiac at 132 bpm and borderline hypotensive at 95/60 mmhg with normal oxygen saturations. The patient looked sick and lethargic with dry mucous membranes but otherwise appeared normal in the exam. Admission labs (Table 1) were significant for leukopenia with white blood count (WBC) of 1.7 (4.5–11.0 K/uL), thrombocytopenia with platelets of 44 K (150–450 K/uL), hyponatremia at 125 (137–145 mmol/L), potassium of 3.7 (3.5–5.1 mmol/L), acute kidney injury with creatinine of 1.9 (0.7–1.2 mg/dL) (unknown baseline), and elevated liver enzymes with AST of 553 (14–36 U/L), ALT of 188 (0–34 U/L), and LDH 1120 (140–280 U/L). The thyroid function test was also normal. The acute hepatitis panel was negative. The urine drug screen was positive for amphetamines and marijuana. The computed tomography (CT) abdomen showed splenomegaly without any other abnormalities. Tick bite labs were sent including Ehrlichia, tularemia and Rocky Mountain spotted fever.

A urine drug screen was obtained for suspicion of drug abuse despite her initial denial. We discussed the findings with the patient, and she later admitted to using both amphetamines and marijuana. This led us to take a detailed social history that revealed an unexpected event. The patient raises horses at her ranch, one whom was in pain. The vet prescribed non-steroidal anti-inflammatory drug (NSAID) pain medication (phenylbutazone) for the horse, and she recognized that the horse had pain relief. The patient was frustrated as naproxen did not help her this time and took phenylbutazone to alleviate her pain. The patient admitted to taking three doses of a 400-pound horse equivalent dose of phenylbutazone about 3 days before the start of her symptoms.

Poison control and toxicology centers were notified but no antidote was recommended. Due to suspected acute liver damage from phenylbutazone, we decided to start an N-acetyl cysteine and sodium bicarbonate drip. She was three IV doses over the course of 21 h, a loading dose with 15 g over one hour, a second dose of 4 g over 4 h, and a third dose 8 g over 16 h. Patient also received two units of cryoprecipitate for suspected disseminated intravascular coagulation (DIC) as her fibrinogen was low at 80. Yet, her international normalized ratio (INR) and peripheral smear were normal. We also initiated empiric doxycycline to cover for tick-borne illness. The patient’s labs (Table 1) improved during her 3 days stay and was clinically feeling better but unfortunately decided to leave the hospital against medical advice before complete recovery occurred.

One week after her discharge, tick titers came back abnormal for Ehrlichia with positive IgM and IgG. Numerous calls attempted to notify the patient but were unsuccessful.

## 3. Discussion

Phenylbutazone, also known as “Bute”, is an equine NSAID which functions as a reversible COX inhibitor, thereby inhibiting prostaglandin H synthesis and prostacyclin and subsequently decreasing inflammation [1]. It was made available in 1949 for use in humans with treatment of ankylosing spondylitis, gouty arthritis, and rheumatoid arthritis, but was withdrawn from the market in the late 1970s due to an increased risk of agranulocytosis. Phenylbutazone has now replaced chloramphenicol as the most common cause of fatal drug-related aplastic anemia and is contraindicated for use in humans [2]. Its active metabolite, oxyphenbutazone, has a mean half-life of 77 h in humans (50–100 h), and is highly protein-bound (98%) in the serum [3]. Given the high protein affinity, it is capable of displacing drugs such as warfarin and phenytoin from their binding sites on albumin, thereby increasing their serum concentrations. It is metabolized in the liver and is excreted by the kidneys [4]. Phenylbutazone is widely available over the counter and is usually sprinkled as powder over the food that the horses will consume.

Most cases of phenylbutazone toxicity are treated similar to other NSAID poisoning with supportive therapy, including airway management, volume resuscitation, and correcting for metabolic acidosis. As with other NSAID toxicities, dialysis has not been shown to be an effective treatment option as it is highly protein-bound [5]. Activated charcoal of 25–100 g in adolescents/adults or gastric lavage have also been used if the patient presents within 1 h of ingestion. Several scales have been developed that attempt to codify causality of drug toxicity into objective criteria [6]. The best-known scale is the Roussel Uclaf Causality Assessment Method (RUCAM) scale, also called the Council for International Organizations of Medical Sciences (CIOMS) scale. Other scales, including the Maria & Victorino scale and the Naranjo scale, are simpler clinical diagnostic scales [7,8,9,10]. While these scales have not been validated in the setting of herbal medications, they may be useful. It is worth mentioning that none of these scales address all risk factors in all patients, and that none are used routinely in clinical practice [7].

Our patient has ingested three doses of phenylbutazone in addition to using naproxen and possibly other over-the-counter NSAIDs. The dose she used was for a 400-pound horse which is much bigger than her weight (170 pounds). Due to all of this, we believe that the patient’s presentation is most consistent with phenylbutazone toxicity leading to aplastic anemia, acute kidney, and liver injury. The long half-life that phenylbutazone exhibits in humans suggests that the medication had been in her blood for two to three weeks, given that medications take around four to five half-lives to be cleared from the body. The level of toxicity could have been further exacerbated by her use of other NSAIDs.

It is worth mentioning that, although her titers for Ehrlichia were positive for both IgM and IgG, we believe that she was exposed to it and her immune system was capable of clearing the infection on its own; therefore, explaining the positivity for both immunoglobulins. Yet, she could have had a mild infection which added symptoms to her clinical presentation. Ehrlichial infection can have a variety of laboratory abnormalities; the most common, occurring in 50 to 90 percent of patients, are leukopenia (often accompanied by a left shift), thrombocytopenia, elevated plasma levels of aminotransferases (transaminases), lactate dehydrogenase, and alkaline phosphatase. Anemia and an elevated plasma creatinine concentration also may be observed [11,12].

## 4. Conclusions

This case is a great example of the importance of taking proper history including social and drug history. Patients may sometimes hide their usage of over-the-counter medications and herbal medicinal preparations from physicians. Multiple medications, such as antibiotics, anti-helminths, and anti-inflammatory agents, are used for humans and animals, but it is not recommended for humans to ingest the formulations made for animals as they have different pharmacokinetics and absorption properties.

## Figures and Tables

**Table 1:** Showing Laboratory data on admission and on hospitalization day 3 with references range.

Laboratory Data	Values on Admission	Values on Day 3	Reference Range
White blood cells	1.7	3.2	4.5–11.0 K/μL
Platelets	44,000	125,000	150–450 K/μL
Sodium	125	139	137–145 mmol/L
Potassium	3.7	4	3.5–5.1 mmol/L
International Normalized Ratio	2.4	N/A	2.0–3.0
Creatinine	1.9	1.1	0.7–1.2 mg/dL
Aspartate Aminotransferase	553	118	14–36 U/L
Alanine Aminotransferase	188	68	0–34 U/L
Fibrinogen	80	N/A	207–442 mg/dL
Lactate Dehydrogenase	1120	300	140–280 U/L
Thyroid Stimulating Hormone	2.13	N/A	0.465–4.68 uIU/mL
Free Thyroxine (T4)	1.2	N/A	0.9–1.7 ng/dL
T3	157	N/A	100–200 ng/dL
Hepatitis Panel	Negative	N/A	Negative
Urine Drug Screen	Amphetamines and Marijuana	N/A	Negative

N/A: Not Applicable.

## Data Availability

Data available upon request.
